# Does that look heavy to you? Perceived weight judgment in lifting actions in younger and older adults

**DOI:** 10.3389/fnhum.2013.00795

**Published:** 2013-11-25

**Authors:** Corrina Maguinness, Annalisa Setti, Eugenie Roudaia, Rose Anne Kenny

**Affiliations:** ^1^School of Psychology, Trinity College DublinDublin, Ireland; ^2^Institute of Neuroscience, Trinity College DublinDublin, Ireland; ^3^The Irish Longitudinal Study on Ageing, Trinity College DublinDublin, Ireland; ^4^School of Applied Psychology, University College CorkCork, Ireland

**Keywords:** action perception, motion perception, visuomotor, sensorimotor, embodied cognition, motor simulation, weight judgment, aging

## Abstract

When interpreting other people's movements or actions, observers may not only rely on the visual cues available in the observed movement, but they may also be able to “put themselves in the other person's shoes” by engaging brain systems involved in both “mentalizing” and motor simulation. The ageing process brings changes in both perceptual and motor abilities, yet little is known about how these changes may affect the ability to accurately interpret other people's actions. Here we investigated the effect of ageing on the ability to discriminate the weight of objects based on the movements of actors lifting these objects. Stimuli consisted of videos of an actor lifting a small box weighing 0.05–0.9 kg or a large box weighting 3–18 kg. In a four-alternative forced-choice task, younger and older participants reported the perceived weight of the box in each video. Overall, older participants were less sensitive than younger participants in discriminating the perceived weight of lifted boxes, an effect that was especially pronounced in the small box condition. Weight discrimination performance was better for the large box compared to the small box in both groups, due to greater saliency of the visual cues in this condition. These results suggest that older adults may require more salient visual cues to interpret the actions of others accurately. We discuss the potential contribution of age-related changes in visual and motor function on the observed effects and suggest that older adults' decline in the sensitivity to subtle visual cues may lead to greater reliance on visual analysis of the observed scene and its semantic context.

## Introduction

Imagine being in a coffee shop and looking at a cup placed on a counter. The cup is completely opaque and you do not know whether it is full or empty. Now imagine your friend reaching for and lifting the cup to move it to another table. By observing the strength of their grip and the speed of their movement, you can immediately deduce that the cup is full, even though you still cannot see what is inside it. What's more, you can also deduce whether they knew that the cup was full or incorrectly expected it to be empty. As such, observing the actions of others involves a form of experience sharing (Brown and Brüne, 2012; Limanowski and Blankenburg, 2013), from which we can derive meaningful information about the agent's intentions and expectations as well as the characteristics of the object acted upon. This information can in turn inform our own interactions with the environment.

Our ability to understand the actions of others (action understanding or action interpretation) is likely mediated by multiple levels of analysis (Grafton and Hamilton, 2007; Thioux et al., 2008), including deducing *how* an action is performed (e.g., with the hand or with the full body), *what* the action is (e.g., lifting a cup) and *why* it is occurring (e.g., to refill the cup because it is empty) (Thioux et al., 2008). The ageing process is accompanied by perceptual and physical changes that may impact the ability to interpret others' actions at these multiple levels of analysis. However, to date, the relationship between ageing, action perception, and judgment of object properties remains relatively unexplored. In younger adults, it has been suggested that the spatiotemporal information derived from action observation engages internal motor simulation of the observed action (Gallese et al., 2004; Knoblich and Sebanz, 2006) and that action understanding and action execution have a shared coding system (Gallese et al., 2004; Knoblich and Sebanz, 2006), as they have been shown to involve overlapping brain regions (Gallese et al., 1996; Rizzolatti et al., 1996). These shared systems may afford our understanding of actions toward objects (Buccino et al., 2004; Hamilton et al., 2006; Ramsey and Hamilton, 2012), as well as intransitive actions such as walking or dancing (Buccino et al., 2001; Calvo-Merino et al., 2005). Although such mechanisms may inform our understanding of *how* and *what* actions are performed, it has been suggested that when people infer the unobservable aspects of the action, such as *why* the action is being performed, they engage an extended network beyond the sensorimotor system to support such “mentalizing” or “theory of mind” processing (Spunt et al., 2011). Other studies have suggested a role for the motor system in conjunction with other brain networks typically involved in theory of mind processing for action interpretation (De Lange et al., 2008; Ramsey and Hamilton, 2012; see also Keysers and Gazzola, 2007).

Motor engagement in action observation is largely modulated by the motor repertoire of the observer (Calvo-Merino et al., 2005). Evidence from healthy and patient populations suggests that spatial awareness of our own and others' body positions (Marzoli et al., 2011, 2013) and sensations arising from our body contribute to interpreting the actions of others (Hamilton et al., 2004; Bosbach et al., 2005; Ní Choisdealbha et al., 2011). For example, when Hamilton et al. (2004) asked participants to judge the weight of a box lifted by an agent while concurrently lifting a box themselves, they noted that the weight of the physically lifted box directly affected perceptual weight judgments. Participants judged the box being lifted by the agent to be heavier when they were physically lifting a light box, and vice versa. In a follow-up study, Hamilton et al. (2006) showed that the magnitude of the bias induced by the motor system on perceptual weight judgments was associated with activation of a specific cluster of visual and motor regions in the brain, leading the authors to suggest that the perceptual and motor systems are not distinct, but interact and influence each other at various levels.

The ageing process is accompanied by declines in motor abilities across a range of tasks. For example, older adults demonstrate differential velocity profiles, decreased fluidity, and increased variability in simple action execution (Cooke et al., 1989; Seidler et al., 2002, for review, see Seidler et al., 2011). The ability to imitate and replicate more complex movement sequences is also negatively affected by ageing (Maryott and Sekuler, 2009; Caçola et al., 2013). Older adults also show declines in the ability to judge the position of their body in space and appear to rely on additional sensory information, largely vision, to compensate for their decline in proprioception (Seidler-Dobrin and Stelmach, 1998; Romero et al., 2003; Barrett et al., 2013). Moreover, Diersch et al. (2012) demonstrated that when online visual information is interrupted, older adults show deficits in predicting the correct time course of action sequences. This indicates that the ability to mentally represent and predict action sequences declines with ageing (see also Saimpont et al., 2009; Gabbard et al., 2011; Diersch et al., 2012). Thus, declines in motor ability with ageing, together with changes in internal forward models of action representation (Diersch et al., 2012), may lead older adults to become more reliant on visual analysis of observed action sequences for action interpretation and inference on object properties. Interestingly, Poliakoff et al. (2010) observed that patients with Parkinson's disease can still perform perceptual weight judgments, however, they may rely more on visual analysis due to declines in the motor system (Poliakoff et al., 2010; Poliakoff, 2013). Thus, while embodied simulation may in part underlie action perception, when we cannot put ourselves “in other people's shoes” through simulation, or when this is not useful to action perception, visual analysis may support action understanding (Brady et al., 2011). Yet, little is known as to how motor changes in non-pathological ageing may affect the interpretation of other people's actions and whether a similar visual strategy may be engaged with advancing age.

Although action execution and action interpretation appear to interact, it is also important to note that they may not bear a direct correspondence. For example, Hamilton et al. (2007) demonstrated that the most reliable physical cues as to the weight of a lifted item do not correspond to the perceptual cues that individuals use when making a weight judgment. Auvray et al. (2011) observed similar discrepancies and suggest that individuals do not engage an “exact copy” of action execution when making perceptual judgments, but rather exploit the most diagnostic visual cues, such as acceleration. Indeed, motion cues such as velocity and acceleration can be used to determine the weight of lifted objects even when visual information is only provided by moving point light displays (Shim and Carlton, 1997). Moreover, the embodied nature of forward models has been questioned, as it has been suggested that motor activation may relate less to “mirroring” or directly matching the actions of others, but rather to anticipating future compatible actions (Csibra, 2007). It has also been suggested that action understanding may be achieved through visual analysis alone without the need for direct embodied simulation (for review, see Giese and Poggio, 2003). This is largely related to our direct visual experience of naturally occurring sequences. The changes that we encounter in action sequences in a natural environment are gradual and are governed by natural laws. Through our constant exposure to naturally occurring sequences, our perceptual system can learn to predict the continuation and outcomes of observed actions (Giese and Poggio, 2003; Perrett et al., 2009). Indeed the spatial and temporal constraints observed in naturally occurring sequences can have a direct effect on our ability to encode (Wallis, 1998; Wallis and Bülthoff, 2001) and, in turn, anticipate the sequence outcome (Perrett et al., 2009). Such visual analysis abilities may be compromised in older adults.

Ageing is associated with deterioration in visual motion perception. For example, older adults are less accurate than younger adults at processing information in biological motion displays (Billino et al., 2008; Pilz et al., 2010; Insch et al., 2012; Legault et al., 2012), suggesting that their ability to process motion cues relevant to action may be impaired. However, age-related declines in motion perception are not limited to biological motion, as other forms of motion perception are also vulnerable to the ageing process (Billino et al., 2008). Older adults are less sensitive at detecting and discriminating the direction of motion in random-dot patterns, a class of stimuli commonly used to address the mechanisms underpinning motion perception (Snowden and Kavanagh, 2006; Bennett et al., 2007; Roudaia et al., 2010; Hutchinson et al., 2012). Older adults are also less sensitive to changes in the speed of moving stimuli (Scialfa et al., 1991; Snowden and Kavanagh, 2006). Thus, age-related declines in visual motion perception may limit older adults' ability to perform visual analysis of observed actions and therefore potentially negatively affect action perception in older adults.

In sum, healthy ageing is accompanied by declines in the ability to perform fine motor movements and declines in visual motion perception, both of which may compromise older adults' ability to interpret other people's actions accurately, either through a reduced ability to extract relevant cues from visual observation and/or through reduced internal simulation of observed actions. In the present study, we examined whether ageing may impact on action understanding by examining the ability of younger and older adults to derive information about the weight of an object, based on the movements of an actor lifting the object. This task is likely to engage aspects of action understanding pertaining to *how* the action is performed (e.g., lifting the box with the hand or with full body motion; the grip and speed of the movements), and *what* the action is (e.g., lifting a small or a large box). It is a naturalistic task with which both younger and older adults have direct experience in everyday life and it is known to provide a reliable measure of sensitivity to interpret the actions of others (Hamilton et al., 2007). Furthermore, the task has been shown to engage both the perceptual and motor systems of the observer (Hamilton et al., 2004, 2007; Poliakoff et al., 2010). Stimuli consisted of a series of videos showing lifting actions of a small box with light weights and a large box with heavy weights. Small box lifts displayed upper limb motion that engaged the forearm and hand and large box lifts displayed the full body motion of the actor lifting the box from the floor. An additional set of videos contained motions that showed the lifting actions of an actor who was told incorrect information about the weight they were about to lift. This deceptive information altered the actors' movement profile, resulting in exaggerated motion that may provide greater visual cues to support weight judgment. The manipulations of box weight category and the actors' movement profile allows for exploration of the relative contribution of visual cues and motor engagement in perceptual weight judgment performance in ageing. For example, although the weights lifted in the large box condition can challenge the ageing motor system via simulation, the perceptual cues pertaining to the weight lifted may be more salient in this condition than in the small box condition (Bosbach et al., 2005). We also collected self-report measures of motor ability (Potter et al., 2009) in the older adult group to assess how perceived motor ability may be related to their capacity to interpret lifting actions.

## Materials and methods

### Participants

Seventeen younger adults (all female) aged 21-28 years (mean age = 24.6 years; *SD* = 1.9 years) and 19 community-dwelling older adults, recruited through an active choral society (18 female) took part in this study. Participation was voluntary and individuals did not receive monetary compensation for their time. Data from two older participants were excluded from the analysis reported below: data from one male participant was removed to maintain consistently with the all-female sample in the younger group and data from one female participant were removed because the participant did not understand the task. The remaining 17 older adults were aged 68-84 years (mean age = 74 years; *SD* = 4.4 years). All younger and older participants reported to be right hand dominant and all reported normal or corrected to normal vision. All participants wore their usual corrective lenses, if needed, at the time of testing. All participants were not suffering from psychiatric or neurological illness by self-report and all provided written informed consent. Our younger and older samples were not strictly matched for years of education, however, older adults had secondary level education or higher and younger adults were college students. The experiments reported here were approved by the St. James Hospital Ethics Committee and conformed to the Declaration of Helsinki.

### Stimuli and apparatus

#### Video stimuli

**Figure 1:**
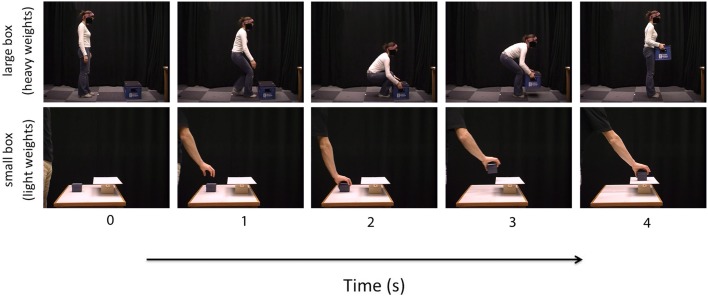
**Illustration of the action sequences in both the small box (lower panel) and the large box (upper panel) perceptual weight judgment trials [static images extracted from the video stimuli provided by Bosbach et al. (2005)]**.

Stimuli were made available by the authors of Bosbach et al. (2005). Stimuli consisted of 8 videos of a male actor lifting a small box and 8 videos of a female actor lifting a large box. The small box videos displayed the right arm and hand of the actor lifting the small box from a table and putting it on a small shelf. The large box videos displayed the full body of the actor lifting a large box from the floor. In all videos, the external features of the box remained constant, but the weight of the box varied (see Figure 1). The small box weighed 50, 300, 600, or 900 g. and the large box weighted 3, 6, 12, or 18 kg. For both the small and large boxes, four non-deceptive videos showed the actor lifting the box after being told correct information about the weight of the box and four deceptive videos showed the actor lifting the box after being told incorrect information about the weight of the box (e.g., lighter than the true weight of the box). All videos showed the actor and the box from the side-view. Each video was approximately 4 s in length and was displayed at a rate of 25 frames per second. Participants viewed the videos at a distance of 60 cm and the images in the videos subtended a visual angle of approximately 14° horizontally and 11° vertically. The experiment was driven by Presentation® software and was presented on a Sony Vaio PC laptop with a 14 inch LCD screen.

#### Perceived motor-efficacy scale for older adults

All older adult participants completed a subset of 19 items taken from the Perceived Motor-Efficacy Scale for Older Adults (Potter et al., 2009). This questionnaire measures the self-reported ability to engage in a number of everyday manual activities and has been shown to relate to actual physical ability. The selected items assess the perceived capability to execute tasks that engage precise manual hand movements and activities that engage full body movements, i.e., activities most relevant to the current experiment. The Appendix contains a list of all administered items. Each item was followed by a 0-10 rating points scale (0 = strongly disagree; 10 = strongly agree).

### Procedure

For the computer-based experiment, participants were seated at a distance of approximately 60 cm from the screen. They were instructed that they would view a number of videos of a person lifting either a small or a large box and that following each video presentation they would be asked to estimate the weight of the box the actor lifted by choosing one of four weight options shown onscreen (50, 300, 600, 900 g. for small boxes and 3, 6, 12, 18 kg. for large boxes). Participants were told that one option was always correct. Participants were offered the choice to view weight options in ounces and pounds and a number of the older adult sample opted for this option. On each trial, the video was presented for 4 s, which was then followed immediately by the response screen. Older participants responded verbally and the experimenter entered their responses by pressing the corresponding button on the keyboard. Younger adults responded by pressing the appropriate button themselves. In all cases, the button press immediately initiated the beginning of the next video. The experiment was presented in four blocks: two blocks contained only non-deceptive videos and two blocks contained both non-deceptive and deceptive videos. The blocks containing only non-deceptive videos were always shown first, however, the order of the small and large box blocks was counterbalanced across participants. In the non-deceptive blocks, each of the four weights was repeated 3 times in random order. In the deceptive blocks, each weight was repeated once in the deceptive and once in the non-deceptive form. Each block was preceded by two practice trials to familiarize the participants with the task. Excluding practice trials, the computer task comprised of 40 trials in total, 24 trials in the non-deceptive blocks and 16 trials in the deceptive block and was approximately 10 min in duration. Following the computer based task, older adult participants completed the questionnaire comprised of the 19 selected items from the Perceived Motor Efficacy Scale for Older Adults (Potter et al., 2009). The experimenter read aloud each item and asked the participant how strongly they agreed with the statement on a scale ranging from 0 (strongly disagree) to 10 (strongly agree). The participant's response was recorded by the experimenter on the sheet. The questionnaire took approximately 5-10 min to administer. Younger adults did not complete the questionnaire, as it is specifically designed to assess perceived motor ability in older adults and as such is not informative for a younger population. All younger adults were active and were not suffering from any mobility impairments.

### Analysis

Data for non-deceptive videos were analyzed using the mean weight estimates, as well as signal-detection measures of sensitivity *(d′)* and response bias (*c*) (Macmillian and Creelman, 2005). Mean weight estimates for each non-deceptive video were calculated by averaging the weights reported in three trials in the non-deceptive block and one trial in the deceptive block. Linear regression was used to obtain the slope and intercept of the best-fit line for each individual's estimated weights as a function of the physical weight of the box, for the small and large boxes separately. In this analysis, accurate perception of the weights would yield a slope of 1 and an intercept of 0, while a slope of 0 would indicate no relationship between perceived and actual weight.

*d*′ scores for discriminating between each pair of adjacent weights were calculated for each participant according to the standard procedure for one-dimensional classification experiments (Macmillian and Creelman, 2005). Cumulative *d*′ scores were then obtained by summing the *d*′ scores for discriminating weights (W) W1 from W2, W2 from W3, and W3 from W4, yielding an overall measure of sensitivity for the small and the large box conditions. The loglinear adjustment method was used to adjust for extreme values of hits and false alarms (Stanislaw and Todorov, 1999). Similar methods were used to obtain the cumulative response bias *(c)* scores for each participant in the small and large box conditions.

Due to the limited number of deceptive trials, it was impossible to calculate *d′* and *c* measures for this condition, therefore, data were analyzed by obtaining the slope and intercept of the best-fit line to the weight estimates for the small and large box condition separately.

Whereas the mean weight estimates, and the fitted regression lines, are contaminated with participants' response bias, the *d′* measure represents an unbiased estimate of the participant's sensitivity for discriminating the weights (Macmillian and Creelman, 2005). The measure of response bias (*c*) was used to determine whether participants showed a preference to use either the higher or the lower end of the weight scale.

Slope and intercept values of the linear regression fits, and *d′* scores were analyzed using separate 2 × 2 mixed-design analyses of variance (ANOVA) with Age (older and younger) as the between-subjects factor and Box Type (small or large) as the within-subjects factor. *c* scores across Age and Box Type were tested against zero using one sample *t*-tests.

## Results

### Non-deceptive trials

**Figure 2:**
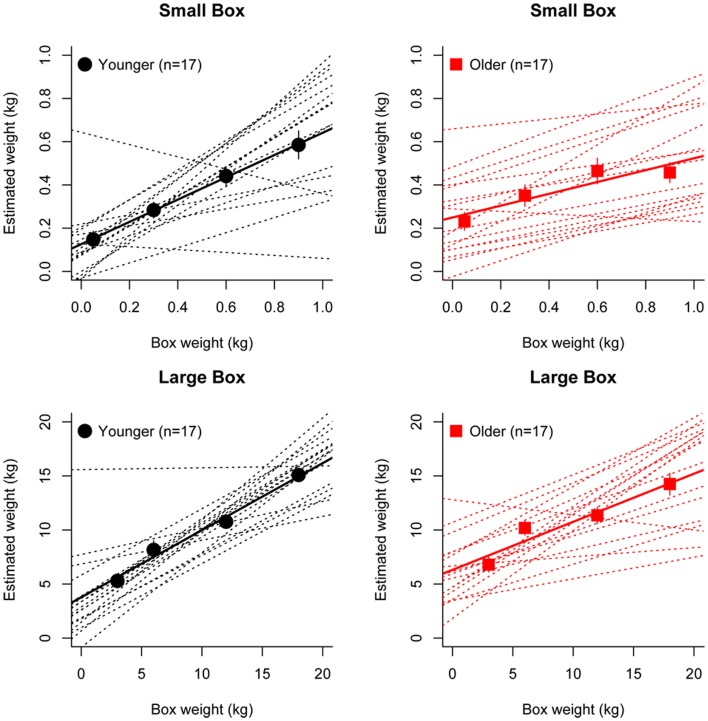
**Linear regression fits to the group average (bold line) and individual (dashed line) perceptual weight estimates for younger (black) and older (red) participants in the small box (upper panel) and large box (lower panel) conditions**.

**Figure 3:**
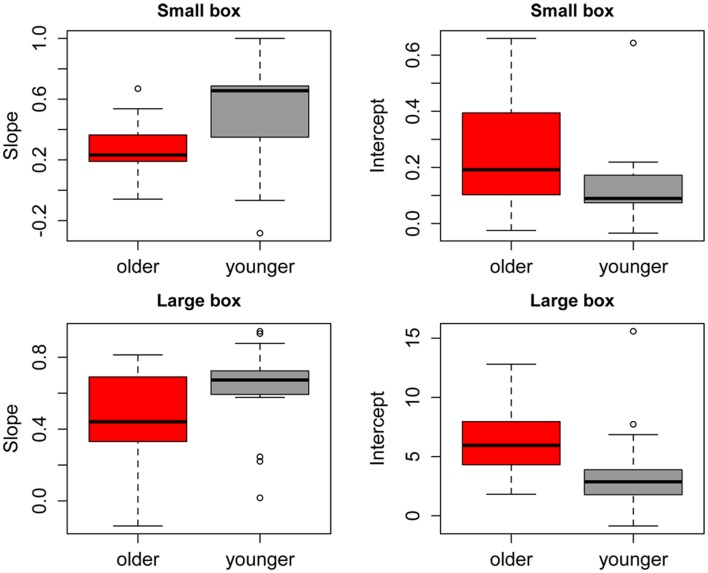
**Mean slopes (left) and intercepts (right) of fitted regression lines for younger (gray) and older (red) participants in the small box (top) and large box (bottom) conditions.** Error bars represent the standard error of the mean.

Figure 2 shows the group average mean weight estimates of younger and older participants for non-deceptive videos in the small and large box conditions, as well as individual subjects' regression line fits. The 2 (Age) × 2 (Box Type) ANOVA on slope values revealed a significant main effect of Age [*F*_(1, 32)_ =8.56, *p* = 0.006], as slopes were shallower in the older group (mean = 0.36) compared to the younger group (mean = 0.57). There was also a significant main effect of Box Type [*F*_(1, 32)_ = 6.43, *p* = 0.02], with shallower slopes in the small box (mean = 0.39) compared to the large box (mean = 0.53) conditions (see Figure 3). There was no significant Age x Box Type interaction [*F*_(1, 32)_ < 1]. The 2 (Age) × 2 (Box Type) ANOVA on intercept values revealed significant main effects of Age [*F*_(1, 32)_ = 5.32, *p* = 0.03], with higher intercepts in the older group compared to the younger group. The main effect of Box Type was also significant [*F*_(1, 32)_ = 71.33, *p* < 0.001], as intercepts in the large box were higher than in the small box. The Age × Box Type interaction was also significant [*F*_(1, 32)_ = 4.4, *p* = 0.04]. Tests of simple main effects revealed a significant effect of Age for the small box [*F*_(1, 32)_ = 4.47, *p* = 0.04; mean younger = 0.13, mean older = 0.25] and the large box [*F*_(1, 32)_ = 4.86, *p* = 0.03; mean younger = 3.78, mean older = 6.31] conditions (see Figure 3). Thus, older participants showed overall shallower slopes and higher intercepts for both small and large box conditions.

#### Sensitivity d′ analysis

**Figure 4:**
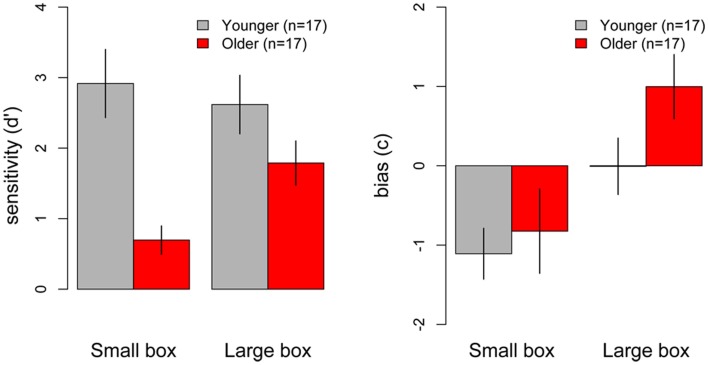
**Mean *d*′ (left) and bias (right) measures for perceptual weight discrimination performance in younger (gray) and older (red) participants across the small and large box conditions.** Error bars represent the standard error of the mean.

Figure 4 (left) shows the mean sensitivity*(d′)* scores for younger and older participants in the small and large box conditions. Higher *d*′ scores represent better discrimination ability. As can be seen in the figure, older participants showed overall poorer sensitivity for discriminating weights than younger participants, especially in the small box condition. A 2 (Age) × 2 (Box Type) ANOVA on *d*′ scores revealed a significant main effect of Age [*F*_(1, 32)_ = 18.61, *p* < 0.001], with younger participants showing overall higher *d*′ scores than older participants. The main effect of Box Type was also significant [*F*_(1, 32)_ = 5.61, *p* < 0.001], with overall higher *d*′ scores in the large box compared to the small box condition. The Age × Box Type interaction was also significant [*F*_(1, 32)_ = 4.55, *p* = 0.04], indicating that the effect of Age depended on the type of box. To decompose the interaction, simple main effects of Age were analyzed for the small and large box separately. Analyses revealed that older participants showed significantly lower *d*′ scores in the small box condition [*F*_(1, 32)_ = 18, *p* < 0.001; younger mean = 2.92, older mean = 0.7], but there was no significant difference between *d*′ scores in the two groups in the large box condition [*F*_(1, 32)_ = 2.5, *p* = 0.12] (see Figure 4). Thus, older participants showed poorer sensitivity than younger participants for discriminating weights in the small box condition, but showed similar performance to younger participants in the large box condition.

#### Bias analysis

Figure 4 (right) shows the mean response bias *(c)* scores for younger and older participants in the small and large box conditions. Positive *c* scores indicate participants' bias for using the upper end of the weight scale (higher weight estimations), negative *c* scores indicate participants' bias to respond at the lower end of the scale (lower weight estimations), and *c* scores near zero indicate no response bias for either end of the scale. To test for the presence of response bias, *c* scores were compared against zero across the small and the large box condition in the younger and older adult groups. In the small box condition, younger participants showed a significant negative bias, with *c* scores being significantly different from zero [*t*_(16)_ = −3.46, *p* = 0.003], however, older participants showed no significant bias, as *c* scores did not differ from zero [*t*_(16)_ = −1.55, *p* = 0.14]. In the large box condition, the pattern was reversed, such that older participants showed a significant positive bias [*t*_(16)_ = 2.46, *p* = 0.03], while younger participants showed no response bias, as their *c* scores did not differ from zero [*t*_(16)_ = −0.02, *p* = 0.1]. Thus, younger participants preferred to use the lower end of the weight scale in the small box condition only, while older participants preferred to use the upper end of the weight scale in the large box condition only (see Figure 4).

#### Deceptive trials

Linear regression was performed on each individual participant data set for the deceptive trials in order to calculate a slope and an intercept value for the small and the large box condition. Slope and intercept values were analyzed separately using a 2 × 2 mixed design analysis of variance (ANOVA), with Age (younger or older) as the between subjects factor and Box Type (small or large) as the within subjects factor. For the slope analysis, no significant main effects of Age [*F*_(1, 32)_ = 2.05, *p* = 0.17]; or Box Type [*F*_(1, 32)_ < 1] were observed. There was no significant interaction between Age and Box Type [*F*_(1, 32)_ < 1]. For the intercept analysis there was no significant effect of Age [*F*_(1, 32)_ = 2.78, *p* = 0.1]. There was a significant main effect of Box Type [*F*_(1, 32)_ = 96.9, *p* < 0.001], with lower intercept values for the small box condition. However, there was no evidence for a significant interaction between Age and Box Type [*F*_(1, 32)_ = 2.44, *p* = 0.13].

#### Perceived motor-efficacy scale for older adults scores

Table 1 shows the average scores from the Perceived Motor-Efficacy Scale broken down into five subscales validated by Potter and colleagues (Potter et al., 2009). All listed item numbers pertaining to the subscales can be viewed in the Appendix. Higher scores in each subscale indicate greater perceived motor ability, with a maximum score of 10. To examine the relationship between perceived motor-efficacy and perceptual weight judgment performance in the current task, we correlated the scores in each different subscale with slope estimates of the linear regression fits obtained in our experiment. There was a significant negative correlation between the Potter et al. (2009) Confidence Indicator (CI) and the slope of the non-deceptive large box condition (*r* = −0.62, *p* = 0.007). The CI is a measure of how cautious or confident someone is in their overall motor ability. This correlation suggests those who were more cautious (i.e., lower CI scores) had higher accuracy in perceptual weight judgments in this condition. There was also a significant positive correlation between the Potter et al. (2009) Perceived Manual Ability (PMA) and the slope of the deceptive small box condition (*r* = 0.57, *p* = 0.02). PMA reflects self-reported ability to use small tools and perform actions related to the use of the hands. Therefore, those with higher PMA scores performed more accurately in perceptual weight judgments in this condition, with their perceived judgments increasing in line with the physical weight of the object. No other correlations between slope measures and motor efficacy scores were found.

**Table 1:** **Mean scores (standard deviations) for each Perceived Motor-Efficacy subscale administered**.

**N17 (Older adults)**		
**Potter subscales**	**Item numbers**	**Mean score (*SD*)**
Perceived motor ability in the face of ageing	7; 16; 27; 3; 4	6.11 (2.28)
Perceived ability to perform precise movements	9; 14; 19; 32; 11	7.51 (2.58)
Perceived motor ability in demanding contexts	37; 15; 23; 24; 33	5.49 (2.52)
Perceived manual ability culturally specific	10; 38	9.26 (0.87)
Confidence indicator	12; 21	3.38 (2.25)

## Discussion and conclusion

Older age brings a number of physical and perceptual changes that can potentially impact older adults' ability to understand other people's actions and the characteristics of the objects acted upon. However, little is known about the effects of ageing on action perception. The present study aimed to fill this gap by using a previously-established paradigm involving weight judgment of objects lifted by an actor (Shim and Carlton, 1997; Bosbach et al., 2005; Hamilton et al., 2007). There are four main findings. First, older participants showed poorer weight estimation than younger participants for all non-deceptive videos, as evidenced by shallower slopes and higher intercepts of the function relating their weight estimates to the physical weight of the object. However, calculating participants' sensitivity (*d*′) for discriminating the different weights revealed that older participants were especially impaired in the small box condition, while performance in the large box condition was equally good in both groups. Thus, light weights were more difficult to discriminate from one another for older adults than for younger adults. Second, we found that response bias differed between older and younger groups, with older participants showing a tendency to use higher weight estimations for weights in the large box condition and younger participants showing a tendency to use lighter weight estimations in the small box condition. Third, younger and older participants showed comparable weight estimation performance in the deceptive small and large box conditions, further indicating that older adults are not impaired in weight estimation when enhanced visual cues are available. Finally, there was a significant positive correlation between two aspects of self-report motor abilities and weight judgment performance, which indicates a relationship between older adults' judgment of weights based on action observation and their own motor abilities.

One previous study of perceptual weight judgment in Parkinson's disease (PD) patients found that only PD patients showed evidence of poor performance, while younger controls and healthy age-matched controls did not show a significant difference in weight estimation performance (Poliakoff et al., 2010). Our current findings, however, suggest that healthy older adults' performance does differ from younger adults, at least for the small box condition. Older participants in the Poliakoff et al. (2010) study were, on average, younger than in the present study, which may have diminished the possibility of finding age-related differences in performance. Participants in that study were also allowed to lift two weights on either end of the scale prior to the experiment, which may have improved their performance.

### Visual cues in action perception

As noted earlier, perceptual weight judgments involve visual analysis of the observed scene and changes in the velocity of movements provide strong diagnostic criteria for accurately deducing the weight of a lifted object (Shim and Carlton, 1997; Hamilton et al., 2007). Overall older adults' performance was worse than that of younger adults, with shallower slopes in weight estimation performance especially in the small box condition, when the weights were light (<1 kg) and the differences between the weights were small (~300 g.). The velocity profiles of the lifting actions in this condition were relatively similar across weights and may have been more challenging for the ageing visual system to exploit. Indeed, motion perception studies have demonstrated that ageing is associated with marked decreases in speed discrimination (Scialfa et al., 1991; Snowden and Kavanagh, 2006). Interestingly, older adults showed similar weight estimation performance to younger adults (as measured by slope estimates) for deceptive trials in the small box condition. This may be due to differences in the available visual cues in the deceptive and non-deceptive videos. In deceptive videos, when the actor is given incorrect information regarding the box weight (e.g., “you are going to lift a light weight” when the weight is heavy), this deceptive information results in online adjustment of the weight lifting behavior. The resulting motion profile increases the ratio of lift phase vs. the reach/grasp phase durations in the deceptive condition relative to the non-deceptive condition (Bosbach et al., 2005). It is possible that older adults are better able to exploit the visual cues in this condition and hence support more efficient performance. However, it also is important to consider that the number of trials in this condition was limited in the current study and these results should be interpreted with caution. Overall, results in the small box conditions suggest that older adults rely heavily on visual cues to judge weight from the actions depicted in the videos.

Consistent with previous studies, perceptual weight sensitivity was greater in the large box compared to the small box condition (Bosbach et al., 2005). For example, Bosbach et al. (2005) demonstrated that participants were more accurate in detecting whether an actor was surprised by the weight of a lifted box in the large relative to the small box condition. They concluded that there are additional and more salient perceptual cues available when full body motion to heavy weights is employed, possibly leading to a better performance in heavy box condition in their study. These more salient visual cues may relate to the velocity information pertaining to the weight lifted in the action sequences. In this condition, the weights were heavy and differences between the weights was also substantial (changes of 3 kg. between weights). Consequently, the differences in the motion profiles of the lifting sequences may have been more salient. Interestingly, we found no differences in weight discrimination of older and younger participants in the large box condition, suggesting that the visual cues available in this condition were sufficiently salient for older adults to exploit. In addition, previous studies have suggested that older and younger adults may rely more on global form information when processing biological motion (Pilz et al., 2010). Unlike the small box condition, the large box condition contained full body motion, which also may have increased the relative importance of global form information in this condition.

In light of a decline in motor ability, it is possible that older adults may become more dependent on visual analysis of the observed action sequence. Indeed, previous findings suggest that individuals with proprioceptive (Bosbach et al., 2005; Toussaint and Meugnot, 2013) and motor disorders (Poliakoff et al., 2010) may engage a visual strategy for the purpose of action understanding. For example, individuals with short term limb immobilization may rely more on visual analysis for tasks which naturally induce internal motor simulation in a normal population (Toussaint and Meugnot, 2013). Our results similarly suggest that a more visual strategy may be adopted with advancing age. Specifically, we observed that sensitivity in detecting the weight of a lifted object increased as a function of the saliency of the visual cues.

In line with our study, previous research involving action perception in older adults has reported a decline in the ability to mentally represent or simulate actions. Older adults show a decline in the ability to accurately predict the timing of perceived actions, possibly due to a difficulty in building internal forward models, especially when visual cues are not always available (Diersch et al., 2012). Such behavioral changes in action perception are also reflected in the differential neural activity seen in the ageing brain during action observation. Functional brain imaging studies have shown that although a similar, yet less lateralized, action observation network is activated in younger and older adults (Diersch et al., 2013), older adults tend to engage additional cortical regions during action perception (Nedelko et al., 2010; Diersch et al., 2013). For example, in a task involving action prediction, Diersch et al. (2013) demonstrated that even when viewing familiar movements older adults tended to recruit additional visual regions of the brain to carry out the task, compared to younger adults. This suggests an overreliance on visual processing for action perception with increasing age. Similarly, differential neural activation patterns have been observed during motor execution (Seidler et al., 2011). Behaviorally older adults also exhibit an overreliance on visual input in movement tasks (Seidler-Dobrin and Stelmach, 1998; Romero et al., 2003; Barrett et al., 2013). This overreliance on visual feedback for motor execution may be modulated by functional and structural changes in motor and somatosensory areas of the brain (review see Seidler et al., 2011).

### Motor simulation in action perception

Although older adults' performance may be modulated to a greater extent than younger adults by the saliency of the visual cues, age-related changes in motor ability may also underlie task performance. Specifically, older adults' difficulty in discriminating between the weights of lifted objects in the small box condition parallels behavioral evidence of marked changes in simple motor behavior (e.g., Romero et al., 2003), possibly arising due to degradation in proprioceptive input with advancing age. Older adults also find it more difficult to detect small differences in the weight of physically lifted objects compared to younger adults (Norman et al., 2009). Weight ratio judgments (i.e., how much lighter is object A compared to object B) become significantly less accurate with ageing (Holmin and Norman, 2012) and the thresholds for accurately detecting such differences are over fifty per cent higher in older, compared to younger adults (Norman et al., 2009). If these behavioral changes are linked to impaired (or imprecise) motor simulation of the same actions, they may at least partly explain the age difference in our task.

We also observed a systematic bias in weight estimation, which may be reflective of the motor system of the observer. Specifically, older adults tended to report that all weights were toward the upper end of the weight scale in the large box condition, but not the small box condition, while younger adults showed a bias to report lighter weights in the small box condition and showed no bias in the large box condition. We can speculate that some form of motor simulation was recruited, as older participants would be expected to experience more difficulty lifting heavier weights, whereas younger participants should be more confident in their abilities with all weights. Interestingly we also observed that older adults' subjective judgment about their action-related skills was reflected in task performance. Specifically, accuracy performance (slope) in the deceptive small box condition correlated positively with older adults' perceived manual ability to use small tools and perform actions related to the use of the hands. Weight estimation in the large box condition was also related to older adults' perceived confidence in movement. Specifically, those who reported being more cautious in carrying out movements, i.e., perceived their own movements to be slower than usual and monitored them more, tended to have better performance in the large box condition than those with higher confidence indicator scores, a score that has been linked previously to physical motor performance (Potter et al., 2009). Potter et al. (2009) noted that higher confidence indicators may be associated with higher minor errors in simple motor execution with advancing age. This may relate to the fact that, while some older adults may experience evolving changes in motor ability, they have yet to revaluate and integrate such declines into perceived abilities (Potter et al., 2009). Therefore, those who were more aware of their motor abilities showed better performance in the current perceptual weight judgment task across the small and large box conditions. However, it must be acknowledged that such findings are based on the self reported motor abilities of the older adult participants. The inclusion of more objective measurements of neuropsychological and physical motor capacity would be of benefit to future studies.

### Conclusion and future directions

The current findings advance our understanding of how action perception is affected by the ageing process. Our results strongly suggest that we become increasingly reliant on robust visual cues to interpret the actions of others with advancing age. One possible consequence of this change is that older adults may be compromised in detecting subtle differences between motion profiles in action sequences, which may carry information about the intention of the actor. For example, a recent study showed that older adults were less sensitive to differences in the timing of interactions between two human characters (Roudaia et al., 2013). The timing of events carries important information about causality (Michotte, 1963). When the events involve human movements, the timing of movements carries important social information, such as deception. Due to such changes, it is possible that the ageing brain may use compensatory strategies for action interpretation. For example, the context in which an action is embedded may become essential for older adults to interpret the action, as it has been shown for younger adults in terms of mapping (Iacoboni et al., 2005) and/or inferring the meaning of others' actions, particularly when the observed actions are not encountered on a regular basis (Liepelt et al., 2008). Recent evidence from an object categorization study shows that the effect of context is more pronounced in older than younger adults (Rémy et al., 2013). It may be the case that a similar effect can be found in action understanding with advancing age.

Although the role of visual cues appears to be a plausible account for the present findings, similar to younger adult studies (e.g., Hamilton et al., 2004), the bias found for heavier weights and the correlation between weight judgment and self-perceived action capabilities in older participants suggests that some level of motor engagement may have affected task performance. Future studies should aim to disentangle the relative contribution of declines in physical and perceptual function on action perception with ageing. Finally, examining action understanding at multiple levels of analysis, including *why* an action is performed, may provide further insight into which facets of action perception remain intact or are negatively affected by the ageing process.

### Conflict of interest statement

The authors declare that the research was conducted in the absence of any commercial or financial relationships that could be construed as a potential conflict of interest.

## Appendix

Subset of items taken from the Perceived Motor-Efficacy Scale. Underlined items are reverse scored.

3. I usually do not attempt complex movements because I find it difficult to perform them well
4. I rarely avoid certain movements in case I fall
7. I do not feel more anxious than I used to when carrying out certain movements
9. I am not very good at activities involving precise manual movements
10. I am likely to have some difficulty using a knife and fork
11. I feel confident at adjusting movements to improve their accuracy or efficiency
12. I do not have to monitor, or keep an eye on my movements, more than I used to
14. I feel I am good at activities involving hand-to-eye coordination, such as catching a ball
15. I believe I would have no problems running for a bus if I had to
16. I rarely worry about climbing up or down stairs
19. I expect to be able to shift smoothly from one movement to another
21. I feel that my movements are slower than they used to be
23. If I were to trip-up, I am confident that I could prevent myself from falling to the ground
24. I am likely to have difficulty walking to the top of a large flight of stairs
27. I expect to be able to learn new movements within a short time
32. I consider myself to be good at activities requiring the precise timing of actions
33. I am confident in my ability to walk a long distance without any difficulties
37. I am not likely to have difficulties getting about outside in the wind
38. I believe I can easily perform the actions required when using kitchen or bathroom taps
